# Low molecular weight protein phosphatase APH mediates tyrosine dephosphorylation and ABA response in Arabidopsis.

**DOI:** 10.1007/s44154-022-00041-6

**Published:** 2022-05-18

**Authors:** Yanyan Du, Shaojun Xie, Yubei Wang, Yu Ma, Bei Jia, Xue Liu, Jingkai Rong, Rongxia Li, Xiaohong Zhu, Chun-Peng Song, W. Andy Tao, Pengcheng Wang

**Affiliations:** 1 Shanghai Center for Plant Stress Biology, CAS Center for Excellence in Molecular Plant Sciences, Chinese Academy of Sciences, Shanghai, 200032 China; 2 Bioinformatics Core, Purdue University, West Lafayette, IN 47907 USA; 3 University of Chinese Academy of Sciences, Beijing, 100049 China; 4 State Key Laboratory of Crop Stress Adaption and Improvement, School of Life Sciences, Henan University, Kaifeng, 475004 China; 5 Department of Biochemistry, Purdue University, West Lafayette, IN 47907 USA

**Keywords:** Abscisic acid, tyrosine phosphorylaiton, phosphatase, kinase, phosphoproteomics, transcriptional regulation, RAF, SnRK2

## Abstract

Low molecular weight protein tyrosine phosphatase (LWM-PTP), also known as acid phosphatase, is a highly conserved tyrosine phosphatase in living organisms. However, the function of LWM-PTP homolog has not been reported yet in plants. Here, we revealed a homolog of acid phosphatase, APH, in Arabidopsis plants, is a functional protein tyrosine phosphatase. The *aph* mutants are hyposensitive to ABA in post-germination growth. We performed an anti-phosphotyrosine antibody-based quantitative phosphoproteomics in wild-type and *aph* mutant and identified hundreds of putative targets of APH, including multiple splicing factors and other transcriptional regulators. Consistently, RNA-seq analysis revealed that the expression of ABA-highly-responsive genes is suppressed in *aph* mutants. Thus, APH regulates the ABA-responsive gene expressions by regulating the tyrosine phosphorylation of multiple splicing factors and other post-transcriptional regulators. We also revealed that Tyr383 in RAF9, a member of B2 and B3 RAF kinases that phosphorylate and activate SnRK2s in the ABA signaling pathway, is a direct target site of APH. Phosphorylation of Tyr383 is essential for RAF9 activity. Our results uncovered a crucial function of APH in ABA-induced tyrosine phosphorylation in Arabidopsis.

**Supplementary Information:**

The online version contains supplementary material available at 10.1007/s44154-022-00041-6.

## Introduction

Protein phosphorylation is one of the most important post-translational modifications in eukaryotic organisms. Phosphorylation affects the protein conformation, activity, localization, turn-over, or/and protein-protein interaction (Edelman et al., 1987; Zulawski & Schulze, 2015). In eukaryotes, protein phosphorylation mainly occurs on three amino acid residues, Serine, Threonine, and Tyrosine. Tyrosine phosphosite only represents a small portion (1–2%) of total protein phosphorylation sites in animals and plants. In animals, some hormone receptors or signal molecules, like insulin and epidermal growth factor (EGF), are protein tyrosine kinases (PTKs), and the tyrosine phosphorylation regulates cell division, proliferation, metabolism, and oncogenesis. In plants, although only about 1600 tyrosine phosphosites were detected out of more than 40,000 phosphosites (Ahsan et al., 2020; Mergner et al., 2020), increasing evidence showed that tyrosine phosphorylation also has a crucial role in the regulation of plant immunity, growth, and hormone signaling (Kameyama et al., 2000; Kim et al., 2009; Jaillais et al., 2011; Nito et al., 2013; Macho et al., 2015; Li et al., 2018; Luo et al., 2020). Though no genes have been identified in plant genomes encoding canonical protein tyrosine kinase, some uncanonical dual specific protein kinases may mediate the tyrosine phosphorylation in plants (Champion et al., 2004; Macho et al., 2015; Zulawski & Schulze, 2015).

About 150 protein phosphatases have been identified in the Arabidopsis genome (Kerk et al., 2008; Moorhead et al., 2009; Mergner et al., 2020). Twenty of them are protein tyrosine phosphatases (PTPs), including 22 dual specific PTPs, one Classic I PTP PTP1, one Class II low molecular weight PTP (LMW-PTP), and one Class IV Asp-based PTP, according to their sequence silimilty to animal PTPs (Kerk et al., 2008; Moorhead et al., 2009; Mergner et al., 2020). It is known that PTP1 interacts with MPK6 and regulates plant immunity (Kerk et al., 2008; Bartels et al., 2009). Dual-specific PTPs, MKP1 and IBR5, participate in plant immunity and development by regulating the MAP kinase cascade. In animals, LMW-PTP, also known as Acid Phosphatase (APase, or ACP) due to acidic optimum pH range from 5 to 6 (Alho et al., 2013). ACP mediates the dephosphorylation of many tyrosine kinase receptors and other signaling molecules involved in signal transduction, which is considered as a signaling hub of cancer hallmarks (Alho et al., 2013; Faria et al., 2021). In plants, to the best of our knowledge, the function of LMW-PTP has not been reported yet.

In this study, we demonstrated that Arabidopsis ACP homolog, APH (AT3G44620), is a functional protein tyrosine phosphatase and participates in ABA signaling. In *aph* mutant, but not in wild type, the global tyrosine phosphorylation is strongly increased upon application of ABA. Several splicing factors and post-transcriptional regulators are identified as the putative targets of APH. Consistently, the ABA highly induced genes showed an impaired expression in *aph* mutant. RAF9, a protein kinase that regulates SnRK2 activation, is also in the list of putative APH target. Mutation of Tyr383 in RAF9 abolishes its kinase activity in vivo. Thus, our results reveal a crucial function of APH in regulating ABA-induced tyrosine phosphorylation in Arabidopsis plants.

## Results

### Arabidopsis APH is a functional protein tyrosine phosphatase

**Fig. 1:**
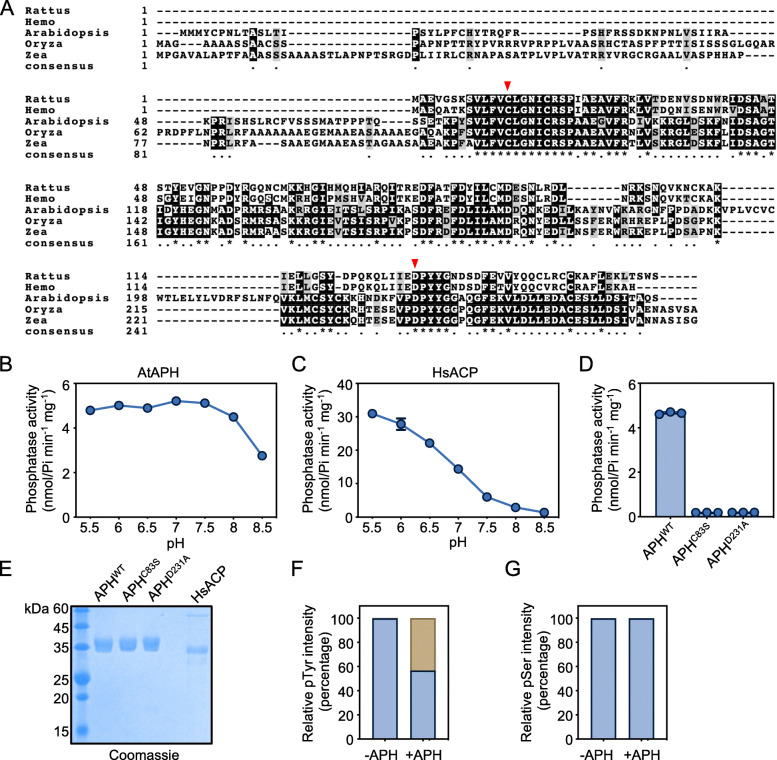
APH is a functional protein tyrosine phosphatase. **A** Sequence alignment of LMW-PTPs in *Rattus norvegicus* (NP_067085.1), *Hemo sapiens* (NP_004291.1), *Arabidopsis thaliana* (NP_001190010.1), *Oryza sativa* (XP_015649938.1), and *Zea mays* (NP_001149867.1). Arrows indicate the conserved Cystine (**C**) and Aspartic acid (**D**) residue in the catalytic domain. **B** The effect of pH on the phosphatase activity of recombinant AtAPH purified from *E. coli*. **C** The effect of pH on the phosphatase activity of recombinant human ACP purified from *E. coli*. **D** Cys83Ser and Asp231Ala mutations abolish APH phosphatase activity. **E** The SDS/PAGE of purified SUMO-HIS-AtAPH and SUMO-HIS-HsACP. **F** The percentage of peptides without (brown) or with (blue) pTyr in the synthetic peptide before and after incubation with recombinant APH. **G** The rate of peptides without (brown) or with (blue) pSer in the synthetic peptide before and after incubation with recombinant APH

Our previous study identified APH (AT3G44620), a homolog of acid phosphatase that was co-immunoprecipitated with PYL1 protein (Wang et al., 2018), suggesting a putative function of APH in ABA signaling. APH is encoded by a single gene in human (*Hemo sapiens*), rat (*Rattus norvegicus*), *Arabidopsis thaliana*, maize (*Zea mays*), and rice (*Oryza sativa*) genome, and the C-terminal catalytic domain of ACPs are highly conserved in different species (Fig. 1A).

Animal Acid Phosphatase is a ubiquitous lysosomal enzyme with an optimum pH range from 5 to 6. To study the function of APH, we first test the pH optimum of recombinant APH purified from *E. coli.* The result showed that Arabidopsis APH, unlike its animal homologs, displayed a broader optimum pH range from 5.5 to 7.5, the activity of APH declines at pH 8.0 and 8.5 (Fig. 1B). Mutation of two conserved residues in the catalytic motif, Cys83 to Ser, or the Asp231 to Ala, abolished the phosphatase activity of APH, respectively (Fig. 1D).

We used two synthetic peptides, EGpSMDTTTETASLLLGMNDSR from SIZ1, and ^375^DKSHNRpYSGV from RAF9, as the substrate to verify the specificity of APH enzymatic activity on phosphotyrosine (pTyr) or phosphoserine/phosphothreonine (pSer/pThr), respectively. As the result, incubation with APH strongly dephosphorylated the pTyr in the RAF9 phosphopeptide (Fig. 1F), but could not affect the abundance of pSer in the SIZ1 phosphopeptide (Fig. 1G). Thus, APH is a functional protein tyrosine phosphatase with an broad optimal pH from 5.5 to 7.5.

### *aph* mutants are insensitive to ABA in post-germination growth

**Fig. 2:**
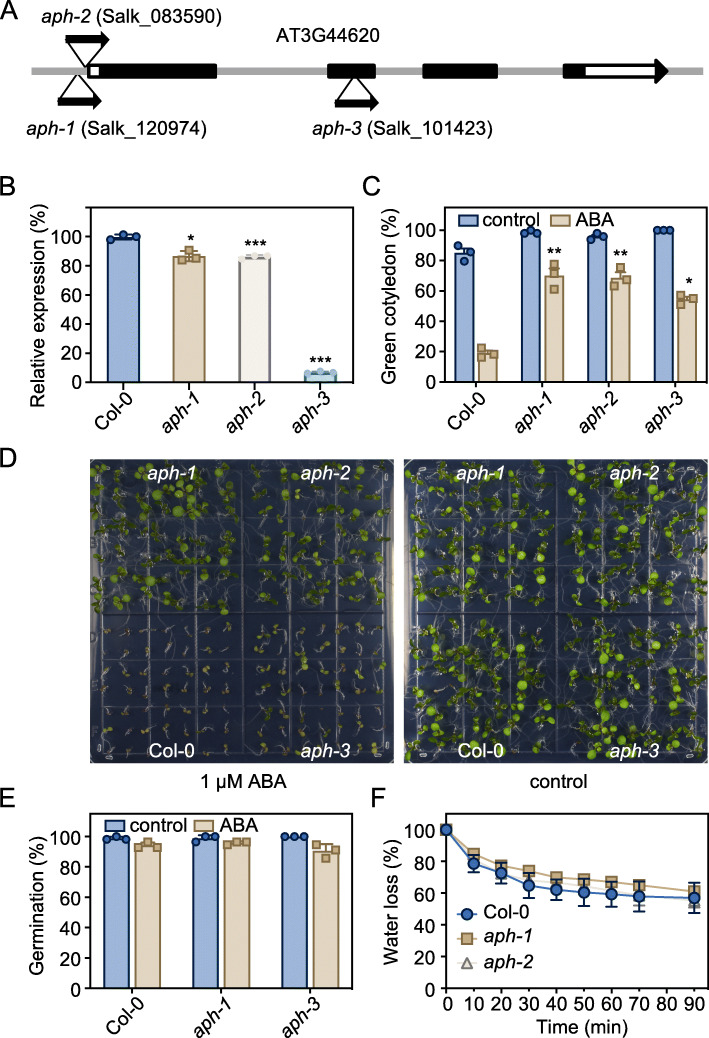
*aph* mutants are insensitive to ABA in the post-germination growth. **A** *APH* gene model and T-DNA insertion sites. **B** qRT-PCR analysis of the *APH* expression in the *aph-1*, *aph-2*, and *aph-3* T-DNA insertion lines. **C** The percentage of seedlings showing green cotyledons after 7 days of germination and growth on 1/2 Murashige and Skoog (MS) medium containing 1 μM ABA. Error bars, SD (*n* = 3). **D** Photographs of seedlings after 10 days of germination and growth on 1/2 MS medium containing 1 μM ABA. **E** The germination rate of seeds after 3 days of germination and growth on 1/2 MS medium containing 1 μM ABA. Error bars, SD (*n* = 3). **F** Water loss rate of the 4-week-old wild type and *aph* mutants. Error bars, SD (*n* = 3). Asterisks indicate significant differences between wild type and *aph* mutants (Student’s *t*-test; *p < 0.05, **p < 0.01, ***p < 0.001)

To study the function of APH, we obtained 3 T-DNA insertion lines *aph-1* (Salk_120974), *aph-2* (Salk_083590) and *aph-3* (Salk_101423) from ABRC (Fig. 2A). The *aph-3* is a null allele, in which the T-DNA is inserted within the second exon, and no expression of *APH* was detected in *aph-3* (Fig. 2B). In *aph-1*, the T-DNA insertion is located in the promoter region of *APH* (Fig. 2A). The RNA sequencing result showed that the T-DNA fragment and the coding region of *APH* formed a fused transcript in *aph-1,* which may result in a truncated or lose-function form of APH (Fig. S1). In *aph-2*, the T-DNA insertion is also located in the promoter region of *APH.* Phenotypic analysis revealed that all the three *aph* mutant lines were insensitive to ABA in the post-germination growth compared to the wild type (Col-0) (Fig. 2C and D, Fig. S2A-C). No difference in the germination rate or water loss was observed between wild type and *aph* mutants (Fig. 2E and F). In addition, overexpression of *APH* does not affect the ABA response in *APH-OE* lines (Fig. S2D). Application of ABA only very slightly (fold change = 1.6, *p* < 0.05) induced the expression of *APH* at later stage.

### Identifying the APH-regulated tyrosine phosphorylation by phosphoproteomics

**Fig. 3:**
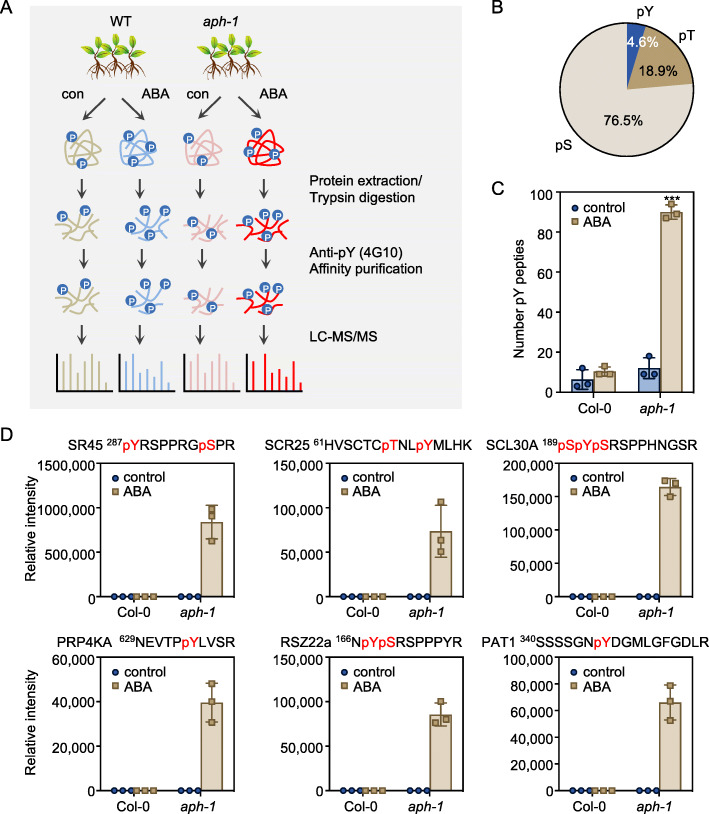
Quantitative phosphoproteomics analysis of global tyrosine phosphorylation in wild type and *aph-1* mutant. **A** Overview of the experimental workflow. **B** The rate of phosphotyrosine (pY), phosphoserine (pS), and phosphothreonine (pT) in the phosphosites identified in this study. **C** The number of pY phosphopeptides identified in wild type and *aph-1* mutant without or with ABA treatment. Error bars, SD (*n* = 3). **D** The tyrosine phosphorylation in some splicing factors and post-transcriptional regulators in the wild type and *aph-1* mutant without or with ABA treatment. The phosphosites identified in these phosphopeptides are highlighted in red. Error bars, SD (*n* = 3). Asterisks indicate significant differences between wild type and *aph-1* mutant (Student’s *t*-test; *** *p* < 0.001)

To dissect the function of APH in ABA signaling, we performed a quantitative phosphoproteomics to compare the APH-mediated tyrosine dephosphorylation in the presence or absence of ABA in the wild type and *aph-1* mutant, the mutant showed the strongest ABA phenotype. As shown in Fig. 3A, phosphoproteomics were conducted with the total protein extracted from wild type and *aph-1* mutant with or without ABA treatment. The tryptic peptides were then subjected to affinity purification using anti-phosphotyrosine (pTyr) antibody 4G10 and subsequently quantified by LC-MS/MS. We detected 16 and 29 pTyr phosphopeptides in the wild-type seedlings without or with ABA-treatment, respectively (Table S1). We also identified 36 and 203 phosphopeptides containing pTyr from the *aph-1* without or with ABA-treatment, respectively. A total of 298 pTyr sites were identified by proteome-scale pTyr enrichment and LC-MS/MS analysis (Table S1). Compared to a total of 6492 phosphosites identified in this phosphoproteomic analysis, 298 phosphopeptides (4.6%) contain at least one phosphorylated tyrosine residue (Fig. 3B). Compared to previous works showing 0.4% (Al-Momani et al., 2018), and 1.8% (Wang et al., 2018) of pTyr in total phosphosites, the 4G10 affinity purification demonstrated a 2.5 to 12-fold enrichment of pTyr peptides.

We then performed a quantitative comparison of the pTyr phosphorylation in the wild type and *aph-1* mutant with or without ABA treatment, using the pTyr sites identified in at least twice out of three biological replicates (Fig. S3A). Eighty pTyr sites corresponding to 76 pTyr proteins were significantly induced in *aph-1* mutant after ABA treatment, compared to the very slight increment of pTyr induced by ABA in the wild-type seedlings (Fig. 3C and S3A). These results suggest that APH may be a negative regulator of ABA-dependent tyrosine phosphorylation, given that dysfunction of APH resulted in an accumulation of pTyr in *aph-1* mutant under ABA treatment.

We further characterized the biological functions of the ABA-induced pTyr proteins in *aph-1* mutant. Fisher’s exact test (*p* < 0.05) was performed to select the biological function of gene ontology (GO) significantly enriched in ABA-induced pTyr proteins in *aph-1* mutant. Only one GO term, mRNA metabolic process (GO: 0016071), is 8.76-fold enriched in the 76 pTyr proteins. The pTyr of some splicing factors like SR45, SCR25, SCL30A, and RSZ22a were only detected in the ABA-treated *aph-1*, but not in control or ABA-treated wild type (Fig. 3D, Table S1). The protein-protein interaction data obtained from the String database also showed that SR45, SCL30A, RSZ22a, and RRP5 are in the cluster of mRNA splicing (Fig. S3B), which is consistent with the result of GO analysis. SR45 is one of the spliceosome components and is involved in pre-mRNA splicing and RNA metabolism. *sr45–1* mutant confers hypersensitivity to glucose and ABA during early seedling growth (Carvalho et al., 2010). PRP4 KINASE A (PRP4KA) is a spliceosome-associated kinase involved in alternative splicing (Kanno et al., 2018). A tyrosine phosphosite pTyr634 in PRP4KA was only detected in ABA-treated *aph-1*seedlings, but not in untreated *aph-1*, nor control and ABA-treated wild type (Fig. 3D, Table S1). The tyrosine phosphorylation of PAT1, a component of the mRNA decapping machinery, also showed a similar *aph-1* dependent pattern to pTyr167 in RSZ22a (Fig. 3D, Table S1). Thus, these results imply that the APH-mediated tyrosine phosphorylation may play an essential role in transcriptional response to ABA.

### Arabidopsis APH regulates transcription of ABA-responsive genes

**Fig. 4:**
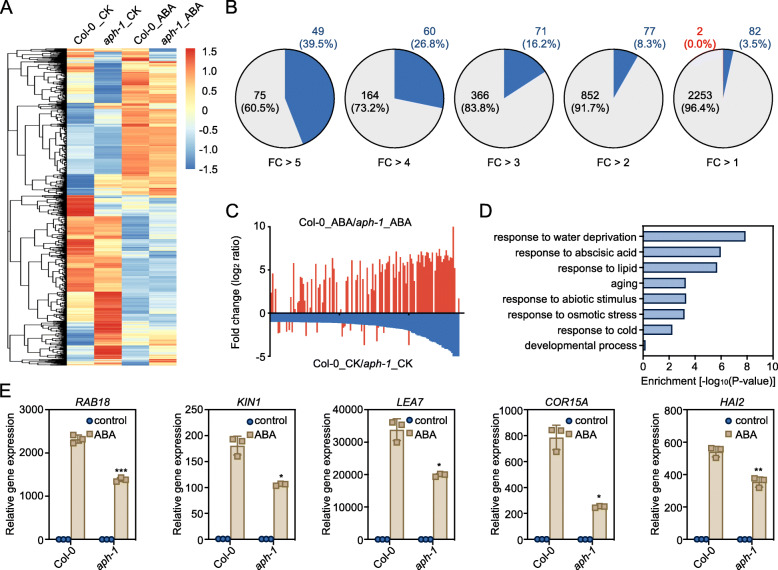
Transcriptome analysis of ABA-responsive genes expression in wild type and *aph-1* mutant. **A** Heat map showing the expression levels of ABA-responsive genes in wild type and *aph-1* seedlings. **B** A major proportion of ABA-induced genes in wild type showed a reduced expression in *aph-1* mutant. DEGs with 5- to 1-fold (*p* < 0.05) are classified according to their expression in response to ABA in Col-0 wild type. Blue, DEGs showed more than two-fold reduction in *aph1* mutant, compared to that in the wild type (FC < 0.5, *p* < 0.05), Red, DEGs showed more than two-fold increment in *aph-1* mutant, compared to that in the wild type (FC > 2, *p* < 0.05). **C** The ABA-responsive patterns of DEGs showed reduced expression (FC < 0.5, *p* < 0.05) in *aph-1* mutant compared to that in the wild type. **D** GO analysis of the DEGs showed reduced expression in the untreated *aph-1* mutant, compared to that in the untreated wild-type seedlings. **E** Expression of the ABA-inducible marker genes in wild type and *aph-1* seedlings upon 6 h of ABA treatment. Error bars, SEM (*n* = 3 biological replicates). Asterisks indicate significant differences between wild type and *aph-1* mutant (Student’s *t*-test; * *p* < 0.05, ** *p* < 0.01, *** *p* < 0.001)

We then performed a transcriptome-sequencing to further explore the role of APH in the regulation of ABA-responsive gene expression. A shown in Fig. 4A and Table S3, a major proportion of ABA highly induced genes showed a reduced expression level in *aph-1* mutant. For example, 124 genes showed at least 5-fold induction by applying ABA in the wild type (Fig. 4B, rightmost panel, and Table S3). Forty-nine (39.5%) out of these 124 genes showed a significant reduced expression (fold change < 0.5, *p* < 0.05) in *aph-1* compared to wild type in the control condition (Fig. 4B, rightmost panel). None of the 124 genes showed increased expression in *aph-1* in the control condition (Fig. 4B, rightmost panel). Thus, most genes that showed reduced expression in *aph-1 *are ABA highly induced genes (Fig. 4C).

GO analysis revealed that these genes with reduced expression in *aph-1*, are mainly involved in water deprivation, ABA, lipid, abiotic stress, cold, osmotic stress, and aging and developmental processes (Fig. 4D and Table S3). Quantitative RT-PCR analysis of several ABA-inducible genes, like *RAB18*, *KIN1*, *LEA7*, *COR15A*, and *HAI2*, showed an impaired induction in *aph-1* (Fig. 4E). Thus, alteration of ABA-responsive genes in *aph-1* supported the function of APH-mediated tyrosine phosphorylation in transcription regulation of ABA response.

### Tyrosine phosphorylation mediates RAF9 activation

**Fig. 5:**
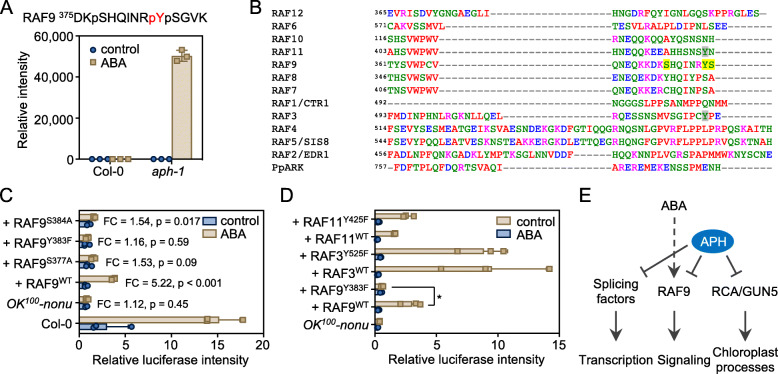
Tyr383 phosphorylation regulates RAF9 activity. **A** The relative abundance of RAF9 phosphopeptide in the wild type and *aph-1* mutant without or with ABA treatment. The tyrosine phosphosite in the phosphopeptide are highlighted in red. Error bars, SD (*n* = 3**). B** Sequence alignment of the peptides in Arabidopsis B2 and B3 RAFs and PpARK/PpCTR1 from *Physcomitrella patens*. The phosphosites showed in **(A)** are highlighted in yellow. The conserved pTyr in RAF3 and RAF11 are highlighted in grey. **C** Activation of the reporter gene by wild type and the non-phosphorylatable mutants of Ser377, Tyr383, and Ser384 in RAF9 in the transient assay in the protoplasts of *OK*^*100*^*-nonu*. Error bars, SEM (*n* = 3 individual transfections). Fold change (FC) and *p*-value (Student’s *t*-test) are indicated. **D** Activation of the reporter gene by RAF3^WT^, RAF3^Y525F^, RAF9^WT^, RAF9^Y383F^, RAF11^WT^, and RAF11^Y425F^, in the transient assay in the protoplasts of *OK*^*100*^*-nonu*. Error bars, SEM (*n* = 3 individual transfections). Asterisk indicates significant differences between wild-type and mutated RAFs (Student’s *t*-test; * *p* < 0.05). **E** A proposed model of APH-mediated tyrosine phosphorylation in the ABA signaling

Recent studies revealed that B2, B3, and B4 subgroup Raf-like protein kinases directly phosphorylate SnRK2s and are required for ABA- and osmotic-induced SnRK2 activation (Saruhashi et al., 2015; Katsuta et al., 2020; Soma et al., 2020; Takahashi et al., 2020; Lin et al., 2021). One phosphopeptide in RAF9, DKpSHQINRpYpSGVK, contains a pTyr site Y383 and two phosphoserine residues pSer377 and pSer384, showed an ABA-induced accumulation in *aph-1* mutant (Fig. 5A and B). To evaluate the role of Tyrosine and Serine phosphorylation in RAF9, we used a transient activation assay in Arabidopsis mesophyll cell protoplasts. The *LUCIFERASE* (*LUC*) reporter gene driven by the ABA-responsive *RD29B* promoter was used as a reporter of ABA response (Wang et al., 2018; Lin et al., 2021). In the wild type, ABA-induced the expression of *RD29B-LUC* in the mesophyll cell protoplasts, while ABA-induced *RD29B-LUC* expression was abolished entirely in the protoplasts of *OK*^*100*^*-nonu*, a mutant lacking nine out of 12 B2 and B3 RAFs (Fig. 5C). Co-expression of wild-type RAF9 partially rescued the ABA-induced expression of *RD29B-LUC* in the protoplasts of *OK*^*100*^*-nonu*. Similarly, co-expression of the RAF9^S377A^ and RAF9^S384A^, the non-phosphorylatable mutations of Ser377 and Ser384 residues, could also partially rescue the ABA-induced expression of *RD29B-LUC* in the protoplasts of *OK*^*100*^*-nonu* mutant (Fig. 5C). Interestingly, co-expression of RAF9^Y383F^, a non-phosphorylatable mutation form of RAF9, cannot rescue the ABA-induced expression of *RD29B-LUC* in the protoplasts of *OK*^*100*^*-nonu* mutant (Fig. 5C). As the tyrosine residue is also existing in RAF3 and RAF11, two other B2 and B3 RAFs, we also introduced non-phosphorylatable mutation Tyr (Y) to Pho (F) in RAF3 and RAF11 and tested the activity of RAF3^Y525F^ and RAF11^Y425F^ mutant forms in the protoplasts of *OK*^*100*^*-nonu* (Fig. 5D). The result showed that the phosphorylation of Tyr525 in RAF3 and Tyr425 in RAF11 is not essential for their activity. Thus, the Tyr383 is a unique phosphosite that is essential for RAF9 function in vivo. Additionally, the phosphorylation of Ser377 and Ser384 is also required for the full activation of RAF9 in vivo.

## Discussion

Although tyrosine phosphorylation only represents a small proportion in total protein phosphorylation, emerging evidence supports a vital role of tyrosine phosphorylation in diverse processes, including immunity, growth/development, and abiotic stress responses in plants (Champion et al., 2004; de la Fuente van Bentem & Hirt, 2009; Macho et al., 2015; Zulawski & Schulze, 2015; Liu et al., 2018). It has been known that tyrosine phosphorylation may have some potential roles in ABA signaling. Application of phenylarsine oxide, an inhibitor of PTP, abolishes ABA-induced stomatal closure (MacRobbie, 2002), and regulates ABA-mediated post-germination development (Reyes et al., 2006). Several dual-specific PTPases, like IBR5 (Monroe-Augustus et al., 2003), PHS1 (Quettier et al., 2006), and AtDsPTP1 (Liu et al., 2015), are known to be involved in ABA signaling. Here we identified APH, a highly conserved tyrosine-specific protein phosphatase, functioning in ABA signaling (Fig. 5E). We demonstrate that APH represses the ABA-mediated protein tyrosine phosphorylation (Fig. 3). Several proteins involved in different mRNA processing steps, including transcription, mRNA splicing, and mRNA decay, undergo ABA-induced tyrosine phosphorylation in *aph-1* mutant. Consistent with this observation, the basal expression of ABA-highly-induced genes is repressed in *aph-1* mutant. APH may mediate ABA-responsive gense expression by dephosphorylating effectors in both transcriptional and post-transcriptional processes. Among putative targets of APH, SR45 has been known to be involved in cotyledon greening (Carvalho et al., 2010). Thus, we reason that the ABA-insensitivity phenotype of *aph* mutants in post-germination growth may be attributed at least in part to the dysfunction of SR45 (Fig. 2).

In addition to transcriptional and post-transcriptional regulation, APH may directly control the function of RAF9, a member of B2 and B3 RAF kinases. Recent studies suggested B2 and B3 subgroup RAFs are essential for ABA- and osmotic stress-mediated SnRK2 activation in Arabidopsis and moss *Physcomitrella patens* (Saruhashi et al., 2015; Katsuta et al., 2020; Soma et al., 2020; Takahashi et al., 2020; Lin et al., 2021). The B2 and B3 have a basal level of activity, which is not induced by ABA (Takahashi et al., 2020; Lin et al., 2021). Such a basal-level activation of RAFs is sufficient to phosphorylate and activate SnRK2s, upon release from PP2C inhibition in the presence of ABA (Lin et al., 2021). In this study, we identified one pTyr site in RAF9, Tyr383, as a putative target site of APH (Fig. 5). The non-phosphorylatable Y383F mutation completely abolished RAF9 activity in the transient assay in the protoplasts. Our results imply that unidentified protein tyrosine kinase(s) may function as an activator to regulate RAF9 activity through phosphorylating RAF9 Tyr383 in ABA signaling. The unknown tyrosine kinase(s) and APH reciprocally coordinate the phosphorylation and function of RAF9. In the future, it would be of great interest to identify the protein kinase(s) that phosphorylate Tyr383 and to dissect how ABA regulates the enzyme activity of APH and unidentified kinase(s). Besides the RAF9 and some proteins involved in transcriptional and post-transcriptional regulation, we also identified dozens of proteins whose tyrosine phosphorylation are up-regulated in *aph-1* (Table S1). These putative APH targets could be a valuable resource for further study of the function of tyrosine phosphorylation in plants.

Although we revealed a crucial role of APH in ABA signaling, how ABA regulates APH activity/function is unknown. The APH was originally co-immunoprecipitated with PYL1 protein. However, we failed to detect the physical interaction between APH and ABA receptor PYR1/PYL/RCAR proteins. In addition, ABA likely does not regulate the APH function through transcriptional regulation, as the expression of *APH* is only very slightly induced by ABA (Fig. S2D). The activity of animal LWM-PTP is regulated by tyrosine phosphorylation and redox regulation. It would be of interest to verify whether Arabidopsis APH also undergoes such post-translational modifications upon ABA signaling. It is also worth noting that the *APH* over-expression lines did not show any observable ABA phenotype (Fig. S2). That is likely because the APH activity is tightly controlled at the post-translational level, or some feedback machinery enhanced these kinases mediating ABA-responsive tyrosine phosphorylation. We also noticed T-DNA insertion in *aph-1* resulted in a fused transcript of the T-DNA fragment and *APH* CDS (Fig. S1). Interestly, *aph-1* showed a stronger phenotype than the “knock-out” mutant *aph-3,* the mechanism is worth to study in the future.

In this study, we used anti-pTyr antibody 4G10-based affinity purification to enrich the phosphopeptides containing tyrosine phosphosites. This method allows us to compare the tyrosine phosphorylation in wild type and *aph-1* (Fig. 3B). However, the specificity and efficiency of this workflow need to be further improved. One of the reasons might be the very low concentration of pTyr in plants. Compared to about 90 typical tyrosine protein kinases in human kinome, no canonical tyrosine protein kinase was identified in Arabidopsis, even though Arabidopsis kinome contains more than 1100 protein kinases (Zulawski et al., 2014). In the future, more efficient and specific strategies, like Src homology 2 (SH2)-domain-derived pY superbinder (Bian et al., 2016), are expected to be helpful for in-depth analysis of tyrosine phosphorylation in plants (Ahsan et al., 2020).

## Materials and methods

### Protein expression, purification, and phosphatase assay

cDNA fragment encoding a truncated APH (aa 57–262) was cloned into *pSUMO vector (LifeSensors).* The resulting plasmids were transformed into *E. coli* BL21 (DE3) cells. The recombinant proteins were expressed and purified using standard protocols. The phosphatase activity was measured using the colorimetric substrate pNPP (Sigma-Aldrich). Reactions were performed in a buffer containing 150 mM NaCl, 2 mM DTT, 1 mM EDTA, 50 mM Tris-Cl/HEPES-NaOH, and the indicated pH from 5.5 to 8.5. Reactions were initiated by the addition of pNPP to a final concentration of 5 mM and terminated by adding 2 M NaOH. The hydrolysis of pNPP was measured by the absorbance reads at 405 nm (A405). The primers used for vector construction and site-directed mutagenesis was listed in Table S4.

For peptide dephosphorylation assay, 500 pmol of synthetic RAF9 peptide DKSHNRpYSGVK and SIZ1 peptide EGpSMDTTTETASLLLGMNDSR (GenScript) were incubated with 100 pmol recombinant in 100 μL buffer (50 mM Tris-HCl, pH 7.5, 150 mM NaCl, 2 mM DTT, 1 mM EDTA) at 37 °C for 2 h. The reactions were quenched by acidifying with 10 μL 10% TFA. After desalted with Sep-Pak C18 tips, the peptides were dissolved in 20 μL 0.2% FA and subjected into LC-MS/MS analysis.

### Phenotyping of germination and post-germination growth

Seeds were surface-sterilized for 10 min in 70% ethyl alcohol, and then rinsed four times in sterile-deionized water. For germination assays, sterilized seeds were grown on medium containing 1/2 MS nutrients, pH 5.7, with or without the indicated concentration of ABA and kept at 4 °C for 3 days. Radicle emergence and green cotyledons were analyzed after placing the plates at 23 °C under a 16 h light/8 h dark photoperiod. Photographs of seedlings were taken at indicated times after transfer to light.

### Water loss measurement

To measure water loss, detached rosette leaves of 4-week-old plants were placed in weighing dishes and left on the laboratory bench with light. Fresh weight was monitored at the indicated times. Water loss was expressed as a percentage of initial fresh weight.

### Protein extraction and digestion

Protein extraction and digestion were performed as previously described (Hsu et al., 2018; Wang et al., 2018). Plants were lysed in lysis buffer (6 M guanidine hydrochloride in 100 mM Tris-HCl, pH 8.5), with 10 mM NaF, EDTA-free protease, and phosphatase inhibitor cocktails (Sigma-Aldrich). Protein lysate was precipitated using the methanol-chloroform precipitation method. Precipitated protein pellets were suspended in digestion buffer (12 mM sodium deoxycholate and 12 mM sodium lauroyl sarcosinate in 100 mM Tris-Cl, pH 8.5) and then were 5-fold diluted. The protein amount was quantified using the BCA assay (ThermoFisher). One mg of protein was then digested with Lys-C and trypsin overnight. The detergents were separated from digested peptides by acidifying using 10% TFA and then centrifuged at 16,000 *g* for 20 min. The digests were then desalted using a 100 mg SEP-PAK C18 cartridge (Waters, Milford, MA).

### 4G10 antibody affinity enrichment

The anti-phosphotyrosine antibody, clone 4G10 (Merck Millipore) was used to enrich the peptides containing phosphotyrosines. About 200 μg tryptic peptides were incubated with 20 μg 4G10 antibody coated on 20 μg Dynabeads (ThermoFisher) at 4 °C overnight, in incubating buffer (50 mM Tris-Cl, pH 7.5, 150 mM NaCl, 0.5 mM EDTA) with phosphatase inhibitor cocktail 3 (Sigma-Aldrich). After washing with washing buffer (50 mM Tris-Cl, pH 7.5, 150 mM NaCl, 0.5 mM EDTA) for 5 times. The bound peptides were eluted with 150 μL of 0.1% TFA, then desalted using a C18 beads StageTip. The Tyr phosphopeptides were dried using a SpeedVac.

### LC-MS/MS analysis

The phosphopeptides were dissolved in 5 μL of 0.25% formic acid (FA) and injected into an Easy-nLC 1000 (Thermo Fisher Scientific). Peptides were separated on a 45 cm in-house packed column (360 μm OD × 75 μm ID) containing C18 resin (2.2 μm, 100 Å, Michrom Bioresources). The mobile phase buffer consisted of 0.1% FA in ultra-pure water (Buffer A) with an eluting buffer of 0.1% FA in 80% ACN (Buffer B) run over a linear 90 min gradient of 6%–30% buffer B at flow rate of 250 nL/min. The Easy-nLC 1000 was coupled online with a Orbitrap Fusion mass spectrometer (ThermoFisher). The mass spectrometer was operated in the data-dependent mode in which a full-scan MS (from m/z 350–1500 with the resolution of 60,000 at m/z 400) was followed by top 10 higher-energy collision dissociation (HCD) MS/MS scans of the most abundant ions with the dynamic exclusion for 60 s and exclusion list of 500. The normalized collision energy applied for HCD was 40% for 10 ms activation time.

### Proteomics data search

The raw files were searched directly against the *Arabidopsis thaliana* database (TAIR10 with 35,386 entries) with no redundant entries using MaxQuant software (version 1.5.4.1) with reporter ion MS2 type. Peptide precursor mass tolerance was set at 20 ppm, and MS/MS tolerance was set at 20 ppm. Search criteria included a static carbamidomethylation of cysteines (+ 57.0214 Da) and variable modifications of (1) oxidation (+ 15.9949 Da) on methionine residues, (2) acetylation (+ 42.011 Da) at N-terminus of protein, and (3) phosphorylation (+ 79.996 Da) on serine, threonine or tyrosine residues were searched. The search was performed with full tryptic digestion and allowed a maximum of two missed cleavages on the peptides analyzed from the sequence database. The false discovery rates of proteins, peptides and phosphosites were set at 1% FDR. The phosphorylation sites induced by ABA treatment in Col-0 and *aph-1* mutant plants were selected using Perseus software (version 1.6.2.1). The intensities of phosphorylation sites were log_2_ transformed, and the quantifiable phosphorylation sites were selected from the identification in at least two replicates in Col-0 and *aph-1* mutant plants. For hierarchical clustering, the intensities of the pTyr phosphorylation sites were first z-scored and clustered using Euclidean as a distance measure for row clustering. The number of clusters was set at 250, with a maximum of 10 iterations and 1 restart. The protein annotation search was performed using the PANTHER database, and enrichment of cellular component was performed using Fisher’s exact test with a cut-off of *p*-value < 0.05. Protein network analysis was performed in the String database (version 11.0) with medium confident score (> = 0.4) as the cut-off, and protein-protein interaction networks were visualized using Cytoscape (version 3.8.2).

### RNA sequencing and data analysis

Total RNA was isolated from two-week-old seedlings of Col-0 and *aph-1* mutant, with and without ABA treatment, using RNeasy Plant Mini Kit (Qiagen). Total RNA (1 μg) was used for library preparation with NEBNext Ultra II Directional RNA Library Prep Kit for Illumina (New England BioLabs, E7765) following the manufacturer’s instructions. Prepared libraries were assessed for fragment size using NGS High-Sensitivity kit on a Fragment Analyzer (AATI), and for quantity using Qubit 2.0 fluorometer (Thermo Fisher Scientific) and KAPA Library Quantification Kit (Kapa, KK4824). All libraries were sequenced in paired-end 150 bases protocol (PE150) on an Illumina Nova sequencer.

The paired-end reads were cleaned by Trimimomatic (Bolger et al., 2014) (version 0.36). After trimming the adapter sequence, removing low quality bases, and filtering short reads, clear read pairs were retained for further analysis. The *Arabidopsis thaliana* reference genome sequence was downloaded from TAIR10. Clean reads were mapped to the genome sequence by HISAT (Kim et al., 2015) with default parameters. Number of reads that were mapped to each gene was calculated with the htseq-count script in HTSeq (Anders et al., 2015). EdgeR (Robinson et al., 2010) was used to identify genes that were differentially expressed. Genes with at least three-fold change in expression with an FDR < 0.05 were considered differentially expressed genes (DEGs).

### Analysis of gene expression by qRT-PCR

Total RNA was extracted from wild type and *aph-1* seedlings treated with 50 mM ABA for 6 h, and isolated using the RNeasy Plant Mini Kit (QIAGEN) according to the manufacturer’s instructions. For real-time PCR assays, reactions were set up with iQ SYBR Green Supermix (Bio-Rad). A CFX96 Touch Real-Time PCR Detection System (Bio-Rad) was used to detect amplification levels. Quantification was performed using three independent biological replicates.

#### Protoplast isolation and transactivation assay

Protoplast isolation and transactivation assays were performed as previously described (Wang et al., 2018). Briefly, protoplasts were isolated from leaves of 4-week-old plants grown under a short photoperiod (10 h light at 23 °C/14 h dark at 20 °C). The *RD29B-LUC* (7 μg of plasmid per transfection) and *ZmUBQ-GUS* (1 μg per transfection), wild type and mutated RAFs (3 μg of plasmid per transfection) were used as an ABA-responsive reporter gene, internal control, and effectors, respectively. After transfection, protoplasts were incubated for 5 h under light in washing and incubation solution (0.5 M mannitol, 20 mM KCl, 4 mM MES, pH 5.7) with or without 5 μM ABA. The primers used form site-direct mutagenesis are listed in Table S4.

## Supplementary Information

**Additional file 1: Fig. S1.** The fused expression of a T-DNA fragment and AT3G44620 exons in *aph-1* mutant. **Fig. S2.** Overexpression of *APH* does not affect ABA sensitivity in *APH-OE* lines. **Fig. S3.** Quantitative comparison of tyrosine phosphorylation in wild type and *aph-1*.

**Additional file 2: Table S1.** The tyrosine phosphosites identified in wild type and *aph-1* mutant, without or with ABA treatment.

**Additional file 3: Table S2.** The DEG in wild type and *aph-1* mutant, with or without ABA treatment.

**Additional file 4: Table S3.** The GO analysis of the down-regulated genes in *aph-1* mutant.

**Additional file 5: Table S4.** Sequences of primers used in this study.

## Data Availability

The RNA sequencing data were deposited to the GEO database with the dataset identifier GSE186664. The phosphoproteomic data were deposited to the ProteomeXchange Consortium via the PRIDE partner repository with the dataset identifier PXD029650. The materials used in this study will be available for research upon request.

## Abbrevations

LWM-PTP: Low molecular weight protein tyrosine phosphatase.
EGF: Epidermal growth factor
PTKs: Protein tyrosine kinases (PTKs)
PTPs: Protein tyrosine phosphatases
ACP: Acid Phosphatase
APH: Arabidopsis ACP homolog
SIZ1: SAP and Miz
GO: Gene ontology
SR45: ARGININE/SERINE-RICH 45
SCL30A: SC35-LIKE SPLICING FACTOR 30A
RSZ22A: RS-CONTAINING ZINC FINGER PROTEIN 22A
PRP4KA: PRP4 KINASE A
LUC: LUCIFERASE
RD29B: RESPONSIVE TO DESICCATION 29B
RAB18: RESPONSIVE TO ABA 18
LEA7: LATE EMBRYOGENESIS ABUNDANT 7
COR15A: Cold-regulated 15A
HAI2: HIGHLY ABA-INDUCED PP2C GENE 2
OK: Osmotic stress activated protein kinase
IBR5: INDOLE-3-BUTYRIC ACID RESPONSE 5
PHS1: PROPYZAMIDE-HYPERSENSITIVE 1
SnRK2: Sucrose nonfermenting 1 (SNF1)-related protein kinase 2 s
PYR1/PYL/RCAR: PYRABACTIN RESISTANCE1/PYR1-LIKE/REGULATORY COMPONENTS OF ABA RECEPTORS
SH2: Src homology 2
BCA: Bicinchoninic acid
EDTA: Ethylene Diamine Tetraacetic Acid
TFA: Trifluoroacetic acid
FA: Formic acid
ACN: Acetonitrile
MS: Mass Spectrometry
HCD: Higher-energy collision dissociation
FDR: False discovery rate
DEGs: Differentially expressed genes
