# Supplementary material for: Low molecular weight protein phosphatase APH mediates tyrosine dephosphorylation and ABA response in Arabidopsis
Source: Stress Biol. 2022 May 18;2(1):23. doi: 10.1007/s44154-022-00041-6 (PMC9345830; doi:10.1007/s44154-022-00041-6)
Supplement: Supplementary file 1 — Additional file 1: Fig. S1. The fused expression of a T-DNA fragment and AT3G44620 exons in aph-1 mutant. Fig. S2. Overexpression of APH does not affect ABA sensitivity in APH-OE lines. Fig. S3. Quantitative comparison of tyrosine phosphorylation in wild type and aph-1. [file 44154_2022_41_MOESM1_ESM.pdf]

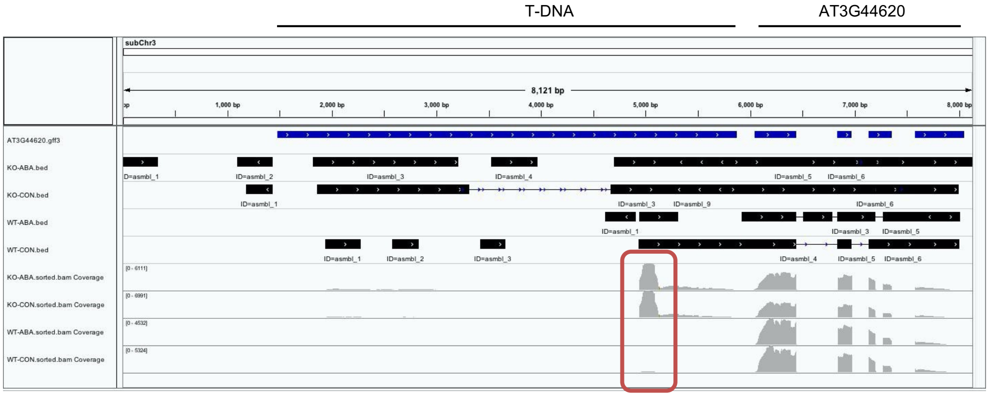

Fig. S1 The fused expression of a T-DNA fragment and AT3G44620 exons in *aph-1* mutant. The expression of a T-DNA fragment was detected in the *aph-1* mutant, but not in the wild type sample, which may result in dysfunction of APH in *aph-1* mutant. The reads from the T-DNA fragment are highlighted.

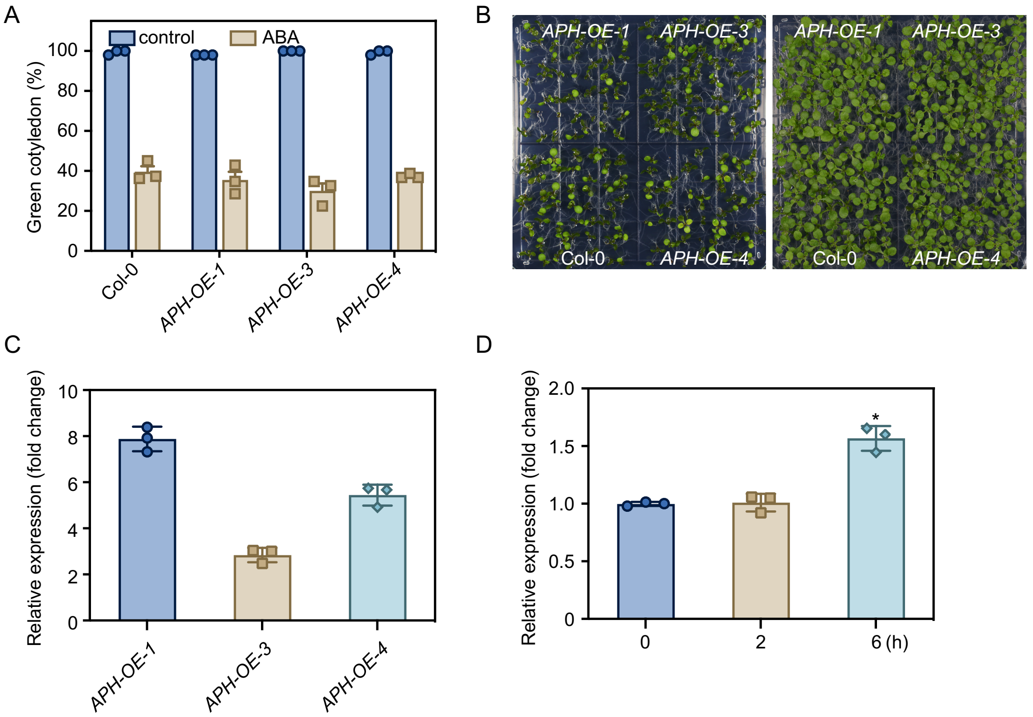

Fig. S2. **Overexpression of *APH* does not affect ABA sensitivity in *APH-OE* lines.**
(A) The percentage of seedlings showing green cotyledons after 7 days of germination and growth on 1/2 Murashige and Skoog (MS) medium containing 1 µM ABA. Error bars, SEM (n = 3). **(B)** Photographs of seedlings after 10 days of germination and growth on 1/2 MS medium containing 1 µM ABA. **(C)** qRT-PCR analysis of the *APH* expression in *APH-OE* lines, related to that in Col-0 wild type. **(D)** qRT-PCR analysis of the *APH* expression in response to ABA in wild type seedlings. Error bars, SEM (n = 3). Asterisks indicate significant differences after ABA treatment (Student's *t*-test; * $p < 0.05$).

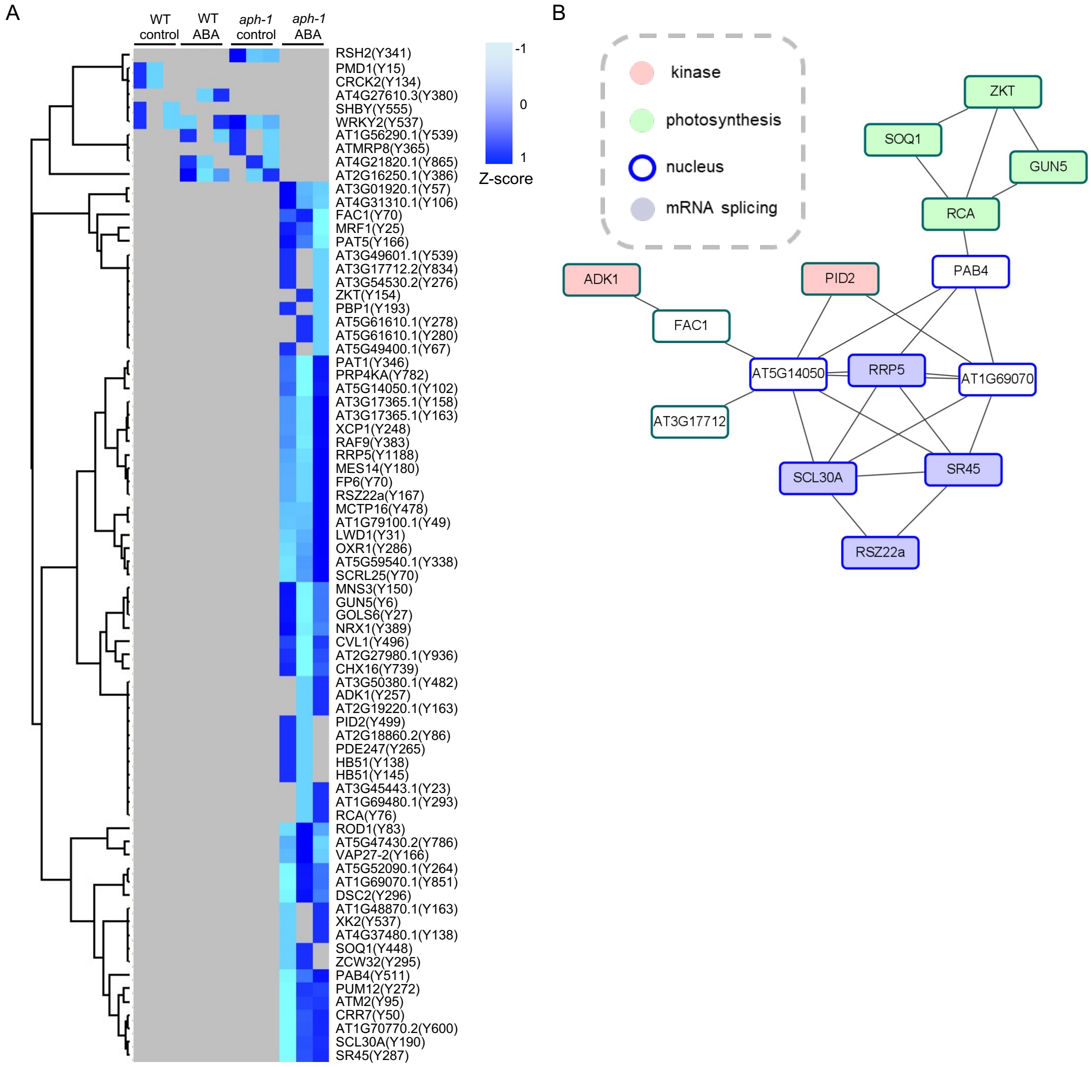

Fig. S3 Quantitative compassion of tyrosine phosphorylation in wild type and *aph-1*. (A) Hierarchical clustering of pTyr sites in three biological replicates in wild type and *aph-1* mutant. (B) String network analysis of ABA-induced pTyr proteins in *aph-1* mutant.
